# Immune correlates of HIV-associated cryptococcal meningitis

**DOI:** 10.1371/journal.ppat.1006207

**Published:** 2017-03-23

**Authors:** Mark W. Tenforde, James E. Scriven, Thomas S. Harrison, Joseph N. Jarvis

**Affiliations:** 1 Division of Allergy and Infectious Diseases, University of Washington School of Medicine, Seattle, Washington, United States of America; 2 Department of Epidemiology, University of Washington School of Public Health, Seattle, Washington, United States of America; 3 Liverpool School of Tropical Medicine, Liverpool, United Kingdom; 4 Institute for Infection and Immunity, St George’s, University of London, London, United Kingdom; 5 St George’s University Hospitals NHS Foundation Trust, London, United Kingdom; 6 Institute of Infectious Diseases and Molecular Medicine, University of Cape Town, Cape Town, South Africa; 7 Botswana-UPenn Partnership, Gaborone, Botswana; 8 Perelman School of Medicine, University of Pennsylvania, Philadelphia, Pennsylvania, United States of America; 9 Department of Clinical Research, Faculty of Infectious Diseases and Tropical Medicine, London School of Hygiene and Tropical Medicine, London, United Kingdom; McGill University, CANADA

## Introduction

**Fig 1:**
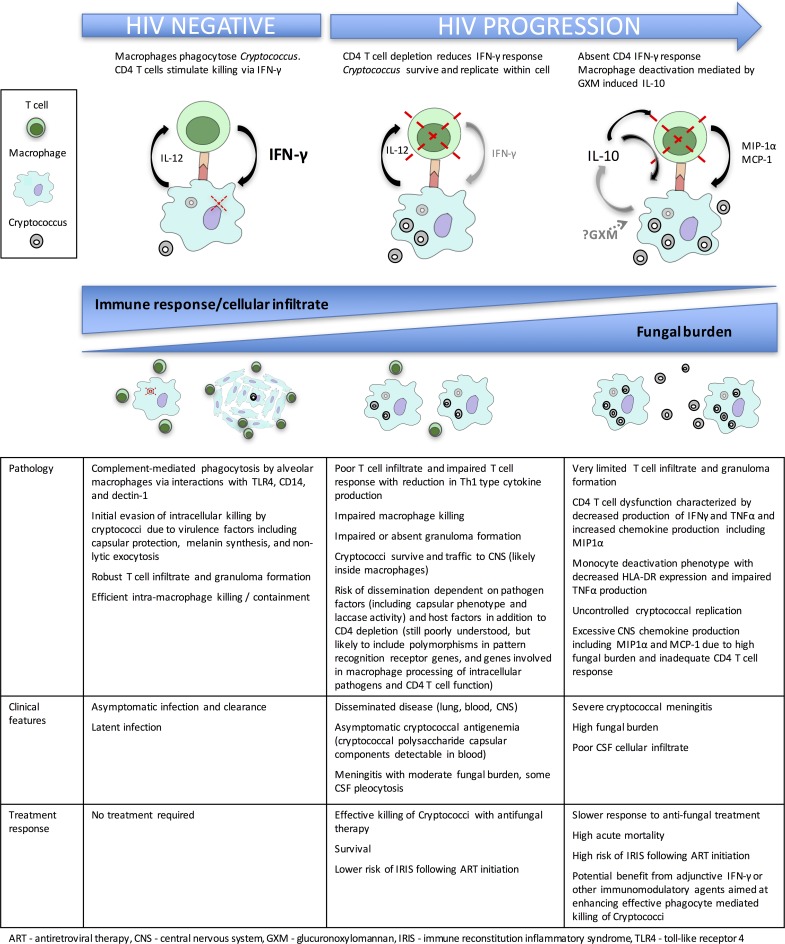
Summary of immune responses to *Cryptococcus* in immune competent hosts and in HIV-infected individuals.

Cryptococcal meningitis (CM) is the leading cause of meningitis in much of sub-Saharan Africa, where it causes up to 20% of all deaths in human immunodeficiency virus (HIV)-infected cohorts [1, 2]. It is primarily caused by infection with *Cryptococcus neoformans*, an encapsulated yeast found widely in the environment. CM may also be caused by *C*. *gattii*, a related organism with a more limited geographic distribution but with the capacity to cause disease in nonimmunocompromised hosts [3]. Following inhalation of spores or desiccated yeast cells, an asymptomatic pulmonary infection occurs in the vast majority of immunocompetent hosts, with detectable antibody responses to *Cryptococcus* protein extract by early childhood [4, 5]. The host immune response leads to clearance of infection or to latent infection with yeast encased within pulmonary granulomata [6, 7]. If the host immune response is impaired, however, yeasts may survive and disseminate through the body hematogenously, resulting in a severe meningoencephalitis (Fig 1) [3].

The host immune response is central to the pathogenesis of cryptococcosis. Not only does an impaired immune response predispose to the condition but variations in the phenotype of the immune response also appear to influence the outcome. Additionally, efforts to reverse the severe immune deficiency through the initiation of antiretroviral therapy (ART) may be complicated by aberrant proinflammatory reactions such as the immune reconstitution inflammatory syndrome (IRIS).

## What is the normal host immune response to *Cryptococcus*?

Alveolar macrophages constitute a primary first-line host defense and recognize *Cryptococcus* spores via Dectin-1 receptors [5]. Phagocytosis occurs through antibody- and complement-mediated, opsonin-dependent pathways and non-opsonin-dependent interaction of cryptococcus surface epitopes with receptors including mannose, dectin-1, CD14, and Toll-like receptor 4 [8, 9]. Once internalized, yeast-containing phagosomes fuse with lysosomes, and intracellular killing may occur under the influence of interferon-γ (IFN-γ) produced by natural killer cells and CD4 T cells [10]. However, in some circumstances and in the absence of appropriate macrophage activation, cryptococci can survive and replicate within the phagolysosome, protected through a variety of mechanisms, including expansion of its thick polysaccharide capsule and laccase-induced melanin synthesis that may neutralize free radicals. Cryptococci eventually exit the macrophage through cell lysis, actin-dependent direct cell-to-cell transfer, or non–lytic exocytosis [11–13]. Parasitism of macrophages facilitate replication and may also facilitate migration of yeast to the central nervous system (CNS) [8, 14].

## What are major susceptibility factors for the development of cryptococcal meningitis in humans?

Cryptococcosis occurs almost exclusively in individuals with impaired cell-mediated immunity. The vast majority of cases worldwide are associated with advanced HIV infection, illustrating the vital importance of CD4 T cells in the human host response [15, 16]. Other risk factors include solid organ transplantation, long-term use of immunosuppressive drugs, diabetes mellitus, rheumatological diseases, advanced liver and renal disease, and hematological malignancies [17]. In addition, a number of rare genetic conditions have been linked to cryptococcosis in non-HIV-infected patients [18].

Despite presumed frequent environmental exposure, not all individuals with deficient cell-mediated immunity, as found in late-stage HIV infection, develop disseminated cryptococcosis. This suggests there may be additional factors that influence host susceptibility. Phagocytic Fcγ receptor 3A 158 F/V polymorphisms have recently been found to be a risk factor for cryptococcal disease, with FcγR3A VV homozygosity conferring higher risk, as have the presence of autoantibodies against granulocyte macrophage colony stimulating factor (GM-CSF) [18–20]. Additional genetic studies comparing DNA from patients with HIV-associated cryptococcal meningitis with that from population and CD4 cell count-matched individuals without evidence of cryptococcal infection are underway. Among exposed individuals, heterogeneity in *Cryptococcus* sequence type, genotype, and phenotype also influence the ability of yeast to disseminate and the clinical outcome [21, 22].

## How does the immune phenotype in HIV-associated cryptococcal meningitis influence outcome?

Proinflammatory and Th1-type immune responses are beneficial among patients with HIV-associated CM, while a lack of immune response is detrimental. Studies from cohorts of patients with HIV-associated CM have shown that higher baseline concentrations of IFN-γ, TNF-α, and IL-6 in the CSF are associated with lower CSF fungal burden, faster fungal clearance on antifungal therapy, and improved survival [23–26]. However, this does not appear to simply be a correlate of a higher CD4 T cell count. Flow–cytometric analyses have suggested that the CD4 T cell phenotype, rather than just the number of circulating CD4 cells, is important in determining the outcome of CM. Following stimulation with cryptococcal mannoprotein, patients with IFN-γ- and/or TNF-α-producing CD4 T cells had corresponding higher CSF lymphocyte counts and CSF cytokine levels, along with lower CSF fungal burden and improved two-week survival odds [27].

Further study has shown that the impaired Th1 response observed in nonsurvivors is not an isolated immune defect but one component of a widespread systemic deactivation of the immune system characterized by monocyte deactivation (decreased human leukocyte antigen [HLA]-DR expression and decreased production of TNF-α following stimulation with lipopolysaccharide); raised IL-10, IL-6, and CXCL10; and increased circulating neutrophils [28]. This immune signature was an independent predictor of two-week mortality and correlated with plasma concentrations of the cryptococcal polysaccharide antigen glucuronoxylomannan (GXM), suggesting that the immune deactivation observed in nonsurvivors may be the result of immunomodulatory actions of *Cryptococcus*. This hypothesis is supported by in vitro studies demonstrating that GXM impairs monocyte activation and antigen presentation and subsequent T cell responses via an IL-10-dependent mechanism [29].

Findings in humans are broadly consistent with animal studies demonstrating protective effects of Th1 immune responses, characterized by IFN-γ production and classically activated macrophages, and detrimental effects of Th2-type responses [30, 31]. However, human studies have not convincingly demonstrated the Th1/Th2 dichotomy found in murine studies. Th2 cytokines (IL-4, IL-13) are less readily detectable in the CSF and are often closely correlated with Th1 cytokines (IFN-γ). [26, 27] This may simply be due to biological differences between species. However, it may also reflect the time course of the immune response. Murine studies typically evaluate the acute immune response following pulmonary inoculation with *Cryptococcus*, whereas human studies represent patients with subacute infection who have a dynamic and evolving host response.

## Does modulation of the immune response improve outcome in CM?

Two main strategies have been adopted to improve host immune response in HIV-associated CM. The first is the use of adjunctive immunotherapy alongside antifungal therapy. IFN-γ has been tested in two phase-2 trials and was found to be safe and associated with a faster rate of CSF fungal clearance. [32, 33] Subgroup analyses suggested that the greatest benefit was gained among patients with a lack of cryptococcus-specific IFN-γ/TNF-α T cell responses. [27]. Neither of these phase-2 trials were sufficiently powered to examine mortality differences; and larger phase-3 trials to determine the mortality benefits of adjunctive IFN-γ therapy, while justified, have not yet been conducted. The second strategy is augmentation of the immune response through earlier initiation of ART. Despite success in the setting of other opportunistic infections [34], this appears not to be beneficial in CM. Two randomized clinical trials have shown increased mortality with early ART. The first showed increased mortality when ART was initiated at 72 hours compared to 10 weeks following CM diagnosis in fluconazole-treated patients [35]. The second, conducted in Uganda and South Africa using amphotericin B-based antifungal therapy, showed increased mortality with ART initiation at 7–13 days compared to 5–6 weeks following CM diagnosis [23]. It was hypothesized that the increased mortality might have been due the development of undiagnosed IRIS.

Immune modulation with adjunctive dexamethasone has also been tested for treatment of CM in a phase-3 trial [36]. This study was terminated early after dexamethasone use was associated with slower fungal clearance from the CSF, higher rates of disability, and a trend toward higher mortality. The immunological mechanisms underlying these findings are not yet known.

## What is cryptococcal immune reconstitution inflammatory syndrome?

IRIS is defined as “a paradoxical deterioration in clinical status attributable to the recovery of the immune system” and in HIV typically manifests as inflammatory reactions at the site of previously treated or previously unrecognized opportunistic infections following the initiation of ART [37]. Cryptococcal IRIS (C-IRIS) is reported to occur in 10%–20% of individuals with HIV-associated CM at a median of 4–9 weeks following ART initiation and has been identified as an independent predictor of mortality [25, 38–40]. The usual presentation is with a recurrence of signs and symptoms of meningitis [41]. Immunological studies have reported a CSF immune response characterized by a CD4 T cell infiltrate, a proinflammatory monocyte phenotype, and increased concentrations of proinflammatory cytokines (TNF-α, IFN-γ, granulocyte colony stimulating factor) [42, 43].

The risk of developing C-IRIS appears to be closely related to the host immune response during the initial episode of CM. Individuals with a lack of inflammation in their CSF during the initial episode of CM, characterized by low CSF white cells (<5/μL), low CSF concentrations of proinflammatory cytokines (IFN-γ, TNF-α, IL-6, IL-8), and increased concentrations of CSF chemokines (CCL2/MCP-1, CCL3/MIP-1α) are at high risk of developing IRIS following ART initiation [43, 44]. Pre-ART cryptococcus-induced IFN-γ production from whole blood is also reduced in those who go on to develop C-IRIS [45]. This paucity of immune response is associated with a higher CSF fungal burden at baseline, slower fungal clearance on antifungal therapy, and increased CSF fungal burdens at the end of antifungal therapy [43, 46]. This increased residual cryptococcal antigen load probably drives C-IRIS during CD4 recovery on ART, with the elevated chemokine levels leading to excessive trafficking of immune cells into the CSF [43, 44, 46–49].

Although no increase in cases of IRIS was observed in the early ART trials, it is probable that the excess mortality was immunologically mediated. The increased mortality associated with early ART was primarily driven by individuals with a CSF white cell count (WCC) <5/γL at randomization—the same group found to be at the highest risk of developing IRIS [23]. Further analysis has shown that early ART was associated with an influx of inflammatory cells into the CSF, with significantly more patients in the early ART arm developing CSF pleocytosis (WCC ≥5/μL) by day 14 (after a median of six days of ART), accompanied by increased CSF concentrations of soluble CD14 and CD163, suggesting increased macrophage activation in the CSF [49].

## Conclusion

Cryptococcal meningitis has emerged as a leading cause of death in individuals with impaired CD4 T cell-mediated immunity. Recent human data suggests that an absence of an effective Th1 response, characterized by IFN-γ and TNF-α production, leads to monocyte deactivation, excessive CNS chemokine production, and poor clearance of infection with increased risk of IRIS and death. Immunomodulation with steroid therapy increases the risk of a poor outcome, whereas augmentation with IFN-γ has demonstrated benefits in faster fungal clearance when coupled with effective antifungal therapy.
